# Age-Based Oocyte Yield in Elective Oocyte Cryopreservation: A Retrospective Cohort Study

**DOI:** 10.3390/diagnostics15172278

**Published:** 2025-09-08

**Authors:** Ronit Machtinger, Atalia Tuval, Ariel Hammerman, Ettie Maman, Ravit Nahum, Raoul Orvieto, Meirav Noah Hirsh, Adva Aizer, Tomer Ziv Baran

**Affiliations:** 1 Department of Obstetrics and Gynecology, Sheba Medical Center, Ramat Gan 52621, Israel; 2 School of Medicine, Gray Faculty of Medical and Health Sciences, Tel Aviv University, Tel Aviv 69978, Israel; 3 School of Public Health, Gray Faculty of Medical and Health Sciences, Tel Aviv University, Tel Aviv 69978, Israelzivtome@tauex.tau.ac.il (T.Z.B.)

**Keywords:** elective oocyte cryopreservation, fertility preservation, IVF, nomogram, age-based counseling

## Abstract

**Background:** Demand for elective oocyte cryopreservation (OC) among healthy women delaying childbearing is rising worldwide. Yet, clinicians and patients often rely on limited or indirect evidence to predict age-specific mature oocyte yield. Robust, real-world benchmarks are needed to guide expectations, estimate live birth potential, and optimize treatment planning. **Methods:** We retrospectively analyzed 400 healthy women aged 30–41 undergoing their first elective OC cycle between 2019 and 2023 at a large, university-affiliated fertility center. Exclusion criteria included infertility, polycystic ovary syndrome, prior ovarian surgery, and other medical indications for OC. All cycles used a standardized GnRH antagonist protocol with an initial gonadotropin dose of 300 IU/day. Only mature (metaphase II) oocytes were cryopreserved. Age-specific percentiles for total and mature oocyte yield were modeled using the General Additive Model for Location, Scale, and Shape (GAMLSS), and nomograms were developed. **Results:** Mean age was 35.7 years (SD 2.3). Median total and mature oocytes retrieved were 13 (IQR 9–19) and 10 (IQR 7–15), respectively. At the 50th percentile, women aged 30, 35, and 40 yielded 20, 14, and 9 total oocytes, with 15, 11, and 6 mature oocytes cryopreserved. Nomograms across percentiles illustrated a consistent, progressive decline in yield with advancing age. **Conclusions:** Age-based nomograms derived from real-world data can offer a clinically relevant tool to estimate the likely oocyte yield per cycle. They can help set realistic expectations, guide the number of cycles needed to meet fertility goals, and support evidence-based, shared decision-making in elective OC.

## 1. Introduction

Oocyte cryopreservation (OC), widely known as egg freezing, is a medical procedure designed to preserve a woman’s reproductive potential by extracting, freezing, and storing her eggs. These cryopreserved eggs can be thawed and fertilized at a later stage, enabling conception when a woman is ready.

Elective OC, also referred to as social or planned OC, represents a specific application of this technology. It is considered an ethically permissible procedure aimed at preventing future age-related infertility [1]. Unlike medical fertility preservation (e.g., for cancer patients), elective OC is typically pursued for non-medical reasons, such as delaying childbearing due to elective circumstances, including the absence of a suitable partner or career considerations.

OC has become an increasingly viable option in fertile women, with uptake rising globally as it allows women who choose to delay childbearing to better align their reproductive plans with their personal, educational, and professional goals [2,3,4,5,6,7].

Among the key factors influencing the likelihood of achieving a live birth with cryopreserved oocytes, the number of mature oocytes retrieved plays a critical role [8]. This required number increases substantially with age due to the declining quality of oocytes. Several studies have reported real-life outcomes or developed age-based predictive models to estimate how many oocytes are needed to maximize the likelihood of successful fertilization and live birth [9,10,11,12,13].

For example, a summary of 15 years from a large urban, university-affiliated fertility center in New York suggested that women under 38 who cryopreserved 20 or more oocytes had approximately a 70% chance of achieving a live birth [14]. Another multicenter study reported that women under 35 needed 15–20 oocytes to achieve a 70–80% chance of live birth, while women over 35 required at least 20 oocytes for a 50% chance [9].

These thresholds provide useful reference benchmarks, but their direct applicability to healthy women undergoing elective OC is uncertain. Importantly, the typical number of oocytes retrieved per cycle at different ages and thus the number of cycles required for healthy elective OC patients to reach these benchmarks remains unclear.

To address this gap, we analyzed real-world data from healthy women undergoing their first elective OC cycle to develop age-based nomograms for both total and mature oocyte yield. Indeed, the association between increasing age and decreased fertility is well established. Therefore, the specific goal of our study was to quantify the association between age and the number of oocytes retrieved in fertility preservation treatments. By doing so, these nomograms not only help set realistic expectations, guide cycle planning, and support evidence-based, shared decision-making in elective OC but also enable women, healthcare providers, and insurers to pre-estimate expected treatment outcomes across different population segments based on age alone without the need for additional tests such as AMH or AFC.

## 2. Methods

### 2.1. Study Population

This retrospective cohort study included women aged 30–41 years who underwent elective OC at a large, tertiary, university-affiliated medical center between January 2019 and December 2023. This age range was chosen because, according to our country’s law, elective oocyte cryopreservation is legally permitted only within this age interval. Data were extracted from electronic medical records and included age, BMI, reproductive history, and baseline FSH. Only first-OC cycle data were included.

### 2.2. Inclusion Criteria

Women were eligible if they:

- Had no known infertility.
- Had a baseline FSH ≤ 10 IU.
- Had a normal transvaginal ultrasound findings.
- Underwent controlled ovarian stimulation using a GNRH antagonist protocol with an initial dose of 300 IU of gonadotropins (the most prevalent protocol in our division for elective oocyte cryopreservation).

### 2.3. Exclusion Criteria

Women were excluded if they:

- Had a diagnosis of polycystic ovary syndrome (PCOS) according to Rotterdam criteria or had no data regarding their PCOS status.
- Had >45 oocytes in the first cycle.
- Had a history of ovarian surgery, endometriosis, or ovarian cysts.
- Had ovarian markers of diminished ovarian reserve.
- Were treated with other dose of gonadotropins.

### 2.4. Treatment Protocol

According to the medical center protocol, ovarian stimulation began with 300 IU of recombinant FSH daily for the first four days. Gonadotropin doses were adjusted based on serum estradiol and follicular diameter. A GnRH antagonist (0.25 mg/day; Cetrorelix, Merck (Darmstadt, Germany) or Ganirelix, Merck Sharp & Dohme (Rahway, NJ, USA)) was introduced when the lead follicle reached 12–14 mm or on day 6. Final oocyte maturation was induced with a GnRH agonist (Decapeptyl 0.2 mg, Ferring Pharmaceuticals, Saint-Prex, Switzerland) 36 h prior to scheduled retrieval. Oocytes were observed and classified post-retrieval. Only mature (metaphase II) oocytes were cryopreserved.

## 3. Statistical Analysis

Data were analyzed using R software (version 4.3.1) and SPSS (version 28). Categorical variables are presented as frequencies/percentages, and continuous variables are presented as mean ± SD or median (IQR).

The number of oocytes retrieved and the number of mature oocytes suitable for freezing was log-transformed, and multivariable linear regression models were applied in order to study the association between women’s age and the number of oocytes retrieved and the number of mature oocytes suitable for freezing while controlling for prior pregnancy and BMI category. The linear regression models were evaluated to meet the assumptions.

Oocyte percentiles by age were modeled using the General Additive Model for Location, Scale, and Shape (GAMLSS), with cubic spline smoothing for log-normal distributions [15]. The Generalized Additive Model for Location, Scale, and Shape (GAMLSS) allows both the location and distributional shape to vary smoothly with age [15]. From a single fitted model, any percentile can be derived, ensuring smooth and coherent nomogram curves. Compared with quantile regression, which estimates each percentile separately and therefore may lead to inconsistencies, the GAMLSS provides a unified and flexible framework for modeling the entire outcome distribution [16,17].

## 4. Results

Of the 721 women who underwent elective OC during the study period, 400 met the inclusion criteria and were included in the final analysis.

The mean age was 35.7 years (SD 2.3); 64% were normal weight (BMI 18.5–24.9 kg/m^2^), 3.8% were underweight (BMI < 18.5 kg/m^2^), 23.0% were overweight (25–29.9 kg/m^2^), and 9.2% were obese (≥30 kg/m^2^). Prior pregnancy, live birth, and induced abortion were reported by 13.5%, 2.8%, and 12% of participants, respectively.

The median stimulation duration was 10 days (IQR 9–11), with a median gonadotropin dose of 3000 IU (IQR 2700–3300). The median number of total oocytes retrieved was 13 (IQR 9–19), and the median number of mature oocytes was 10 (IQR 7–15).

Multivariable analysis showed that after controlling for prior pregnancy and BMI category, each year of age decreased the number of oocytes retrieved by 8.0% (95%CI 5.4–10.6%, *p* < 0.001) and the number of mature oocytes suitable for freezing by 7.7% (95%CI 4.9–10.3%, *p* < 0.001).

**Figure 1:**
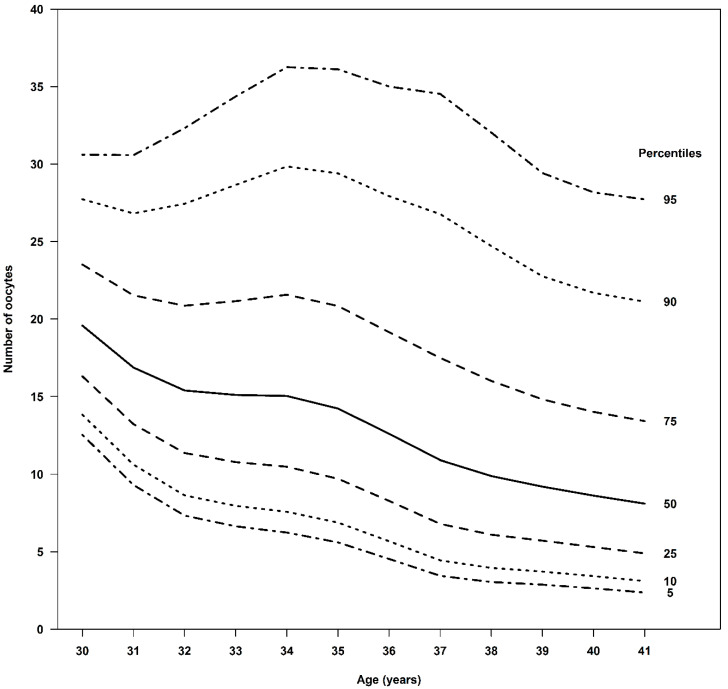
The total number of oocytes per age and various percentiles.

**Figure 2:**
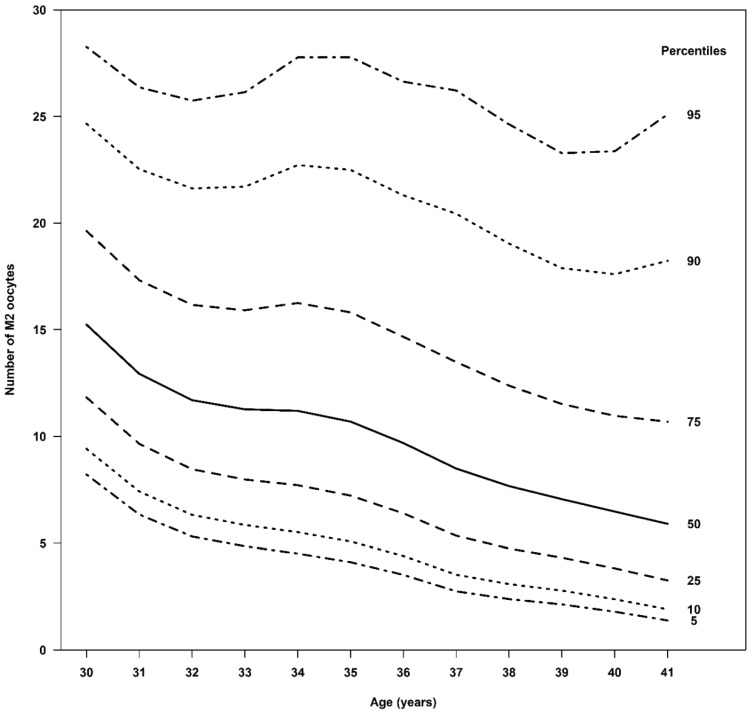
The number of frozen mature oocytes according to age and various percentiles. A nomogram that describes the relationship between the age of women seeking fertility preservation and the distribution of the number of mature oocytes suitable for freezing that were retrieved. The nomogram depicts percentiles 5, 10, 25, 50, 75, 90, and 95. Using the nomogram, it is possible to estimate the range of the expected number of oocytes and, hence, to estimate in advance the number of treatments required to accumulate a sufficient number of oocytes for future fertilization. This nomogram allows the woman to understand the expected range of mature oocytes to be retrieved.

Age-specific analysis revealed a clear inverse association between age and oocyte yield. The 50th percentile of retrieved oocytes for women aged 30, 35, and 40 years was 20, 14, and 9, respectively, while the 50th percentile of mature oocytes suitable for freezing was 15, 11, and 6. The 25th percentile for retrieved oocytes was 16, 10, and 5, with the 25th percentile for mature oocytes being 12, 7, and 4. The 75th percentile for retrieved oocytes was 24, 21, and 14, with the 75th percentiles for mature oocytes being 20, 16, and 11, respectively. Nomograms illustrating these percentiles are provided in Figure 1 and Figure 2 and described in Table 1 and Table 2.

We present a nomogram that describes the relationship between the age of women seeking fertility preservation and the distribution of the number of oocytes retrieved. The nomogram depicts percentiles 5, 10, 25, 50, 75, 90, and 95. This nomogram allows a woman to understand the expected range of oocytes to be retrieved and adjust her expectations from the treatment, even before meeting with the fertility doctor.

Table 1 shows the number of oocytes retrieved by age and percentiles. According to the table, it is possible to understand the number of oocytes retrieved for a certain segment of women at each age. For example, half of women (50%) at age 35 had between 10–21 oocytes retrieved (percentiles 25–75), and 90% of women at this age had between 6 and 36 oocytes retrieved (percentiles 5–95).

Table 2 shows the number of mature oocytes suitable for freezing retrieved by age and percentiles. According to the table, it is possible to understand the number of mature oocytes retrieved for a certain segment of women at each age. For example, half of women (50%) at age 35 had between 7 and 16 mature oocytes suitable for freezing retrieved (percentiles 25–75), and 90% of women at this age had between 4 and 28 mature oocytes suitable for freezing retrieved (percentiles 5–95). These data are important both for women to understand the outcomes of the treatment they will undergo and for healthcare providers and insurers. This assessment can improve the estimation of the expected cost.

## 5. Discussion

Female fertility undergoes a natural decline with advancing age, a process that accelerates notably after the mid-30s, becoming particularly rapid beyond 37 years [18]. This decline is primarily attributed to two interconnected factors: a reduction in the overall quantity of oocytes (diminished ovarian reserve) and a concurrent increase in the rate of aneuploidy, or chromosomal abnormalities, stemming from deterioration in oocyte quality. This inevitable decline in both oocyte quantity and quality leads to a reduced likelihood of natural conception and elevated rates of pregnancy loss. For women aged 40 and above, assisted reproductive technologies (ART) offer limited success, and the probability of achieving a live birth with one’s own oocytes becomes negligible after age 45.

Elective OC is increasingly accepted as a proactive option for women seeking to manage their reproductive timelines. However, the decision to undergo OC can be complex, influenced by various personal, emotional, elective, and financial factors.

Women opt for elective OC for a variety of reasons, including career planning, educational attainment, health status, and current partnership situation [19,20,21,22]. OC offers a means of mitigating the impact of the biological clock, thereby providing women with greater autonomy over their reproductive choices.

Recent trends underscore the growing relevance of this option. In the United States, the annual number of OC cycles increased dramatically over the past decade, rising by approximately 880% between 2010 and 2016 [3], with further uptake observed during the COVID-19 pandemic. Several factors likely underlie this expansion, including shifts in attitudes toward family and career in the post-pandemic era, broader employer-sponsored coverage, the growing availability of clinical outcome data on OC, and diminishing elective stigma [23].

A recent meta- analysis found that the mean age at the time of cryopreservation was 38.1 years (±0.4). The average rate of women returning to thaw their cryopreserved oocytes was 11.1% (±4.7) and the mean age at the time of thawing was 41.8 years (±1.2), with an average interval of 3.8 years (±1.1) between cryopreservation and thawing. The post-thaw oocyte survival rate averaged 78.3% (±5.4). Among those who returned for thawing, the mean number of oocytes originally cryopreserved was 12.6 (±3.6) [24].

The cryopreservation process itself involves several steps, including hormonal stimulation through self-administered injections, frequent clinic visits for monitoring, and the oocyte retrieval procedure, which carries potential risks such as bleeding and infection.

A critical distinction in fertility preservation is between the quantity of oocytes retrieved (yield) and their inherent quality. While a higher number of retrieved oocytes is generally correlated with improved live birth rates, it is imperative to recognize that oocyte quality holds equal, if not greater, importance for successful fertility outcomes. Oocyte quality, which pertains to the potential of a fertilized oocyte to result in a live birth, is predominantly influenced by a woman’s age. This highlights that simply maximizing the number of retrieved eggs without considering their biological integrity and developmental potential is insufficient.

Given these complexities, it is crucial that women considering elective OC receive reliable, evidence-based information to make well-informed decisions, set realistic expectations, and effectively weigh the potential benefits and risks [25]. Such information supports women in navigating the emotional aspects of the process—balancing hopes for the future with the possibility of disappointment [26]. In contrast, limited information or unrealistic expectations may lead to regret, emotional distress, or feelings of guilt following the decision [27].

A recent prospective, cross-sectional survey in 278 graduate women investigated their knowledge of fertility general issues and specifically OC. These women demonstrated only moderate fertility general knowledge (64% correct responses). While 93.9% had heard of OC, only 7.2% had considered using it. Formal education was the most common source of fertility information (87.1%), gynecologists were the primary source for decision-making about fertility (85.3%), and the media was the predominant source for OC information (63.4%). Only 26.6% reported feeling well-informed about fertility. Most participants valued fertility (74.9%), yet many indicated they would postpone childbearing until establishing a career (85.2%) and a relationship (85.2%). Half perceived an elective stigma associated with OC, and 70.1% believed that the media conveys an overly optimistic view of motherhood after age 40. This study concluded that, overall, professionally oriented women tend to receive accurate fertility information from formal and clinical sources but rely on the media for fertility preservation information, indicating persistent gaps in fertility planning knowledge [19].

Another observational study included 133 women who underwent elective OC, identified that the absence of a male partner and concerns about age-related fertility decline were the two major motivations for pursuing OC. However, despite receiving comprehensive, personalized counseling, many women did not seem to have a realistic understanding of reproductive aging. Nearly 60% of women overestimated the chances of natural conception, as well as the success of IVF, at the age of 40 years [28]. In another survey of 124 women who received elective OC counseling at a major fertility center, it was found that older women faced more challenges when making decisions about OC. Additionally, women who perceived themselves as more fertile than others of the same age, as well as those with higher levels of decisional conflict, were less likely to pursue OC [29]. All of these findings emphasize the importance of age-specific counseling, particularly for women approaching the end of their reproductive years. The data unequivocally demonstrate that age has a compounding negative effect on fertility preservation outcomes: not only does the quantity of retrieved oocytes decrease with age, but the quality of each oocyte also declines significantly.

Besides elective and clinical issues, the cost of OC is a significant barrier to its broader accessibility. Elective OC for non-medical reasons, while offering a valuable option for fertility preservation, is often not publicly funded and typically requires substantial out-of-pocket expenses that can limit its accessibility for many individuals. These may include costs for the treatment cycle, ovarian-stimulation medications, the day procedure, anesthesia fees, and annual storage fees. Moreover, a single cycle is often insufficient to gather an adequate number of eggs, especially for older women, meaning that multiple cycles may be required. Beyond the per-cycle cost, when a woman is ready to use her cryopreserved eggs, there are additional costs for thawing the eggs, performing in vitro fertilization (IVF), and transferring the resulting embryo [8,30].

Financial constraints may delay the initiation of treatment or discourage some women from pursuing OC altogether, as the costs can appear unaffordable for many individuals. This underscores the need for counseling physicians to also consider the financial aspects of the procedure and to guide patients in making cost-effective, well-informed decisions. Usually, women opting for OC need several egg retrieval cycles to reach an optimal number of frozen oocytes. Given that each OC cycle is billed separately, accurate, age-specific information regarding the minimum and optimal number of oocytes needed to achieve a realistic chance of pregnancy and the estimated number of cycles required to reach this goal is crucial from both clinical and economic perspectives. While the age-related decline in female fertility is well established, the novelty of our study lies in quantifying this decline in terms of expected oocyte yield per cycle in healthy women undergoing elective oocyte cryopreservation (OC). Our presented nomograms provide age-specific estimates of the number of oocytes retrieved and the number of mature oocytes by age per cycle, thereby allowing women and physicians to anticipate not only the clinical effort but also the financial burden of multiple OC cycles. By linking the nomogram-derived estimates of oocyte numbers with the expected number of retrieval cycles, women can better anticipate cumulative costs, enabling more informed financial and reproductive planning.

To support the alignment of clinical, emotional, and financial expectations, our study aimed to develop user-friendly, age-based nomograms to predict the number of total and mature oocytes likely to be retrieved during a first OC cycle in healthy women without known diminished ovarian reserve.

It is well known that freezing the oocytes at a younger age and the accumulation of more mature oocytes result in a better success rate of live birth [7,31,32]. Our findings further underscore the profound impact of a woman’s age at the time of cryopreservation on the quantity of retrieved oocytes. In our cohort, 50% of women aged 30, 35, and 40 had 16–24, 10–21, and 5–14 oocytes retrieved, with 12–20, 7–16, and 4–11 of those oocytes matured and frozen, respectively. The nomograms based on these data can serve as a valuable tool for both patients and clinicians, facilitating more informed and personalized counseling. Counseling must provide realistic expectations regarding success rates, which are heavily dependent on the age at freezing and the number of mature oocytes thawed. Given the clear age-based thresholds for the number of oocytes needed to achieve a reasonable chance of live birth and the average yield per retrieval cycle, counseling must explicitly address the potential necessity of multiple retrieval cycles, particularly for women in their mid-to-late 30s and beyond, to accumulate a sufficient number of oocytes to achieve their desired family size. By helping women align expectations prior to initiating the process, our developed nomograms may also enhance confidence and clarity in decision-making.

In order to provide more practical guidance on how the nomograms can be used in counseling, we specifically link our percentile estimates to published data on oocyte number thresholds associated with live birth probabilities. For example, Goldman’s model [12] indicates that women at the age of 36 who accumulate 18 mature oocytes achieve ~80% live birth probability. In our cohort, a 36-year-old woman at the 50th percentile yielded ~10 mature oocytes per cycle, implying that two cycles may be required to approach this threshold. Similarly, women aged 40 years averaged ~6 mature oocytes per cycle, suggesting that 8 cycles are typically necessary to achieve a comparable probability (Table 3).

This table combines published thresholds for the number of mature oocytes associated with ~80% live birth probability from a previously published model of Goldman et al. [12], with data from our cohort. For each age, the left column shows the number of oocytes required, and the adjacent columns indicate the estimated number of treatment cycles needed to accumulate this number, based on different yield percentiles (5th, 25th, 50th, 75th, 95th). These estimates provide practical guidance for counseling patients regarding the likelihood of achieving clinically meaningful oocyte numbers within one or multiple cycles of elective OC.

By combining our age-based percentiles with these established outcome data, clinicians can provide patients with realistic expectations regarding the likely number of cycles required. This counseling is especially important in the context of financial cost, time, and emotional burden.

Although our study is retrospective, thus inherently limiting causal inferences, its strengths include a relatively large sample size and its setting at a high-volume, tertiary, university-affiliated medical center, which enhances the generalizability of the findings. However, several limitations should be noted.

First, the analysis was limited to women aged 30–41, in line with the eligibility criteria for elective fertility preservation set by the Israeli Ministry of Health [8]. While this age range likely represents the majority of women seeking elective OC, it may not capture outcomes for younger or older patients. Second, anti-Müllerian hormone (AMH) levels, an ovarian reserve marker, were unavailable for most participants, limiting our ability to analyze its association with oocyte yield. AMH is a valuable tool primarily for predicting the quantity of oocytes that can be retrieved following controlled ovarian stimulation; however, in Israel, AMH testing is not covered by public health insurance and requires out-of-pocket payment, leading to its underuse. Nevertheless, recent evidence suggests that AMH may not reliably predict fertility potential, as pregnancies and live births can still occur in women with low AMH levels [33,34,35,36]. Moreover, the American College of Obstetricians and Gynecologists (ACOG) does not currently recommend the use of AMH for fertility counseling in women without infertility diagnoses [37]. Thus, although the lack of AMH prevented sensitivity analyses, our age-based nomograms remain clinically useful in real-world settings where AMH is often unavailable. We highlight the need for future research to integrate AMH data into predictive models but emphasize that age continues to be the strongest determinant of both oocyte yield and quality. Third, our analysis included only patients that underwent elective OC and excluded women with PCOS, prior ovarian surgery, or medical indications for fertility preservation. This decision was intentional, to generate benchmarks specific to healthy women pursuing elective OC. Inclusion of such populations would have introduced heterogeneity: women with PCOS often yield supra-physiologic oocyte numbers, whereas women with prior ovarian surgery or medical comorbidities typically exhibit reduced ovarian reserve. Accordingly, our findings may not be generalizable to these groups and should be interpreted in the context of healthy, non-infertile populations. Future studies are warranted to validate and extend these nomograms in broader clinical settings.

Fourth, our study population may reflect a selection bias, as only women with the financial means to pursue elective OC were included. Because treatment is self-funded in Israel, this group may not fully represent the general population, potentially limiting the socioeconomic generalizability of our findings.

Fifth, the study did not include long-term follow-up on actual live birth outcomes from cryopreserved oocytes. While oocyte yield is a critical intermediate marker, the ultimate measure of success is live birth. Future prospective studies linking age-based oocyte yield to cumulative live birth outcomes are needed to strengthen counseling and refine prognostic models.

Finally, the study included only the first OC cycle for each woman and utilized a single stimulation protocol. Outcomes may vary in subsequent cycles or with different protocols.

In our protocol, the starting dose of 300 IU of gonadotropins may be considered relatively high compared with the doses commonly employed in assisted reproductive treatments. However, in a comparative analysis that evaluated treatment parameters among women undergoing elective OC versus those undergoing IVF, it was found that the starting doses and stimulation durations for elective OC were consistent with those used in our cohort [38].

In summary, age stands as the most influential factor determining both the quantity (yield) and quality of oocytes retrieved in elective OC cycles, profoundly impacting subsequent live birth rates. Effective patient counseling is paramount. It must extend beyond general success rates to provide age-stratified quantitative data on oocyte yield and the number of oocytes required for realistic live birth probabilities.

As the field continues to evolve, ongoing research is essential to gather more robust, age-stratified data, particularly concerning the long-term outcomes and the optimal number of oocytes needed for desired family sizes, to further refine counseling practices and improve patient outcomes.

## 6. Conclusions

Elective OC is an effective method for preserving fertility. However, there is a recognized lack of high-quality evidence to definitively counsel patients on the optimal number of oocytes or retrieval cycles required to achieve a single live birth. The nomograms developed in this study provide a practical tool for estimating the expected oocyte yield from a single oocyte retrieval cycle in healthy women according to age. These nomograms can help women and physicians anticipate not only the clinical effort but also the financial burden of multiple OC cycles. This alignment of clinical and economic expectations supports more informed reproductive planning and allows patients to better assess the affordability of elective OC. Nevertheless, the nomograms should be regarded as preliminary guides rather than substitutes for individualized medical consultation and personalized fertility counseling, which remain essential to establish realistic expectations regarding outcomes and the likelihood of utilizing cryopreserved oocytes. Future research is needed to validate our findings and explore broader applications.

## Figures and Tables

**Table 1:** A heat map presenting the total number of oocytes retrieved by age and percentile.

Age (Years)	Centiles												
	5	10	20	25	30	40	50	60	70	75	80	90	95
**30**	**13**	**14**	**16**	**16**	**17**	**18**	**20**	**21**	**23**	**24**	**25**	**28**	**31**
**31**	**9**	**11**	**12**	**13**	**14**	**15**	**17**	**18**	**20**	**22**	**23**	**27**	**31**
**32**	**7**	**9**	**11**	**11**	**12**	**14**	**15**	**17**	**20**	**21**	**23**	**27**	**32**
**33**	**7**	**8**	**10**	**11**	**12**	**13**	**15**	**17**	**20**	**21**	**23**	**29**	**34**
**34**	**6**	**8**	**10**	**10**	**11**	**13**	**15**	**17**	**20**	**22**	**24**	**30**	**36**
**35**	**6**	**7**	**9**	**10**	**11**	**12**	**14**	**16**	**19**	**21**	**23**	**29**	**36**
**36**	**5**	**6**	**7**	**8**	**9**	**11**	**13**	**15**	**17**	**19**	**21**	**28**	**35**
**37**	**3**	**4**	**6**	**7**	**8**	**9**	**11**	**13**	**16**	**17**	**20**	**27**	**35**
**38**	**3**	**4**	**5**	**6**	**7**	**8**	**10**	**12**	**14**	**16**	**18**	**25**	**32**
**39**	**3**	**4**	**5**	**6**	**6**	**8**	**9**	**11**	**13**	**15**	**17**	**23**	**29**
**40**	**3**	**3**	**5**	**5**	**6**	**7**	**9**	**10**	**13**	**14**	**16**	**22**	**28**
**41**	**2**	**3**	**4**	**5**	**5**	**7**	**8**	**10**	**12**	**13**	**15**	**21**	**28**

**Table 2:** A heat-map presenting the number of frozen mature oocytes by age and percentile.

Age (Years)	Centiles												
	5	10	20	25	30	40	50	60	70	75	80	90	95
**30**	**8**	**9**	**11**	**12**	**13**	**14**	**15**	**17**	**19**	**20**	**21**	**25**	**28**
**31**	**6**	**7**	**9**	**10**	**10**	**12**	**13**	**14**	**16**	**17**	**19**	**23**	**26**
**32**	**5**	**6**	**8**	**8**	**9**	**10**	**12**	**13**	**15**	**16**	**18**	**22**	**26**
**33**	**5**	**6**	**7**	**8**	**9**	**10**	**11**	**13**	**15**	**16**	**17**	**22**	**26**
**34**	**5**	**6**	**7**	**8**	**8**	**10**	**11**	**13**	**15**	**16**	**18**	**23**	**28**
**35**	**4**	**5**	**7**	**7**	**8**	**9**	**11**	**12**	**14**	**16**	**17**	**22**	**28**
**36**	**4**	**4**	**6**	**6**	**7**	**8**	**10**	**11**	**13**	**15**	**16**	**21**	**27**
**37**	**3**	**4**	**5**	**5**	**6**	**7**	**8**	**10**	**12**	**13**	**15**	**20**	**26**
**38**	**2**	**3**	**4**	**5**	**5**	**6**	**8**	**9**	**11**	**12**	**14**	**19**	**25**
**39**	**2**	**3**	**4**	**4**	**5**	**6**	**7**	**8**	**10**	**12**	**13**	**18**	**23**
**40**	**2**	**2**	**3**	**4**	**4**	**5**	**6**	**8**	**10**	**11**	**12**	**18**	**23**
**41**	**1**	**2**	**3**	**3**	**4**	**5**	**6**	**7**	**9**	**11**	**12**	**18**	**25**

**Table 3:** Estimated number of mature oocytes required for ~80% live birth probability (Goldman et al. [12]) and corresponding number of elective OC cycles needed to achieve this goal, based on age-specific oocyte yield in our cohort.

Age (Years)	Number of Mature Oocytes Needed for 80% Live Birth (at Least One Live Birth)	Number of Elective OC Cycles Needed According to the Centiles of Oocytes Retrieved				
		5	25	50	75	95
≤35	14	3	2	2	1	1
36	18	5	3	2	2	1
37	23	8	4	3	2	1
38	27	14	6	4	3	2
39	32	16	7	5	3	2
40	44	22	11	8	4	2
41	55	55	14	10	5	3

## Data Availability

The data presented in this study are available on request from the corresponding author due to privacy.
